# Hemato-oncological outpatient care in medical education: a German pilot-project

**DOI:** 10.1007/s00432-025-06198-7

**Published:** 2025-05-20

**Authors:** Marie Forster, Sophie Winkler, Martin R. Fischer, Dirk Hempel, Valeria Milani

**Affiliations:** 1 University Hospital, LMU Munich, Munich, Germany; 2 Institute of Medical Education, LMU University Hospital, LMU Munich, 80336 Munich, Germany; 3 Dept of Gynecology and Obstetrics and CCC Munich, LMU University Hospital, Munich, Germany; 4 Onkomedeor, Oncological Cancer Center, Fürstenfeldbruck, Germany; 5 ZIO, Center for Integrative Oncology, Zurich, Switzerland

**Keywords:** Outpatient setting, SARS-CoV-2 pandemic, Hematology and oncology, Interprofessional, Teaching, Mentorship

## Abstract

**Purpose:**

To describe the experience of online and practical medical teaching in the outpatient hemato-oncological primary care setting and to analyze challenges and chances for students and teachers in specialized outpatient institutions.

**Methods:**

The study involving medical students of the Ludwig-Maximilians-University (LMU) in their 6th–7th semester evaluates content and didactic methodology of online teaching seminars, bed-side clerkships and mentoring regarding one selected oncological center. The data was collected via questionnaires using Likert-scaled items.

**Results:**

In one outpatient cancer center a total of 279 students attended the online lessons (2020–2023). 102 evaluations were collected, and all aspects of teaching of the online seminars and clerkship were rated very well (mean score of 1.2 on the item “overall evaluation” with a small range of 1.0–1.3, n = 102). Criticism was mainly leveled at technical issues (n = 16). The evaluations (n = 10) of the students attending a one-day bed side teaching revealed high interest in learning the practice in the outpatient setting. 90% stated an improvement in understanding of outpatient practice as well as intersectoral processes due to the one-day bedside teaching and favored an integration of this new teaching format into the regular medical curriculum. Two students applied for a four-week internship and six chose a mentorship in hematology-oncology, resulting in four medical thesis projects in this field.

**Conclusion:**

Increased participation of outpatient centers in medical education improved knowledge on outpatient medicine and interprofessional care and generated interest in the field of oncology. Outpatient cancer specialists should be more involved in the curriculum of medical students.

**Supplementary Information:**

The online version contains supplementary material available at 10.1007/s00432-025-06198-7.

## Introduction

Germany has the most expensive health care system in the European Union, due, among other things, to high costs in the inpatient sector (Korzilius 2019). These costs could be greatly reduced through a stronger relocation of medicine and patient care in the outpatient sector. To this aim, the current government has planned to strengthen the outpatient medical sector in the coalition agreement, e.g., by remunerating certain medical services with the same fee, regardless of whether they are provided in a private practice or in a hospital (Osterloh and Ambulantisierung 2022).

Moreover, the dramatic shift of care towards ambulatory rather than inpatient setting, in particular in the field of hematology and oncology, is also due to the exponential development of new therapeutic agents that do not require hospitalization like oral targeted therapy or immunotherapy. In Germany non-academical outpatient care centers driven by oncology/hematology specialists with full reimbursement allow a nationwide, high-quality cancer care and cover about 40% of all hemato-oncological patients (www.winho.de).

With regard to medical education, a stronger integration and promotion of the outpatient sector has already been stipulated in the “Masterplan 2020” and the new Medical Licensing Regulations (NKLM), planned from 2025 and still under intense political debate (Forschung 2020; Richter-Kuhlmann and Medizinstudium 2025). Several problems in teaching arise for physicians working in the outpatient sector. Long journeys for lecturers or students as well as high-cost pressure and time stress in the daily routine make it difficult to hold courses. As a consequence, students gain significantly more insights into inpatient care during their studies. The inpatient cases are often more critically ill and thus not representative of common case presentation.

Furthermore, medical education in the ambulatory care setting and interaction with health professionals provides a valuable learning experience that contributes to the understanding of interprofessional practice (Saunders et al. 2019). Thus, the evaluation of intersectoral teaching systems is important to support the development of medical teaching from university hospitals and teaching hospitals to teaching practices in oncology.

In response to the SARS-CoV-2 pandemic, medical education faculties have quickly transitioned the entire curriculum to online formats. All aspects of medical education were affected, and many challenges were experienced by trainees and potentially compromised their core training—including their mental health (Kohls et al. 2021; Zis, et al. 2021). Social distancing and deferral of clinical rotations had a high impact in providing authentic patient experiences as key component of medical education (Rose 2020; Kaul et al. 2021). However, there were several opportunities for innovations, creativity and research that contributed to the advancement and transformation of medical education (Loda et al. 2020; Herrmann-Werner, et al. 2021; Oliveira Franco et al. 2019; Becker et al. 2020).

The purpose of this study is to describe the experience on medical teaching by faculty members from outpatient or distant institutions starting from the pandemic with online teaching and furthermore to describe feasibility for students to practice in an outpatient cancer center setting with focus on a selected center and to finally analyze challenges and chances for both teachers as well as for medical students.

## Methods

### Study design and population size

This observational study started with the SARS-CoV-2 pandemic in the winter term of 2020 up to summer term 2023 involving faculty members as well as medical students of LMU Munich in their 6th and 7th semester, section hematology/oncology.

**Fig. 1:**
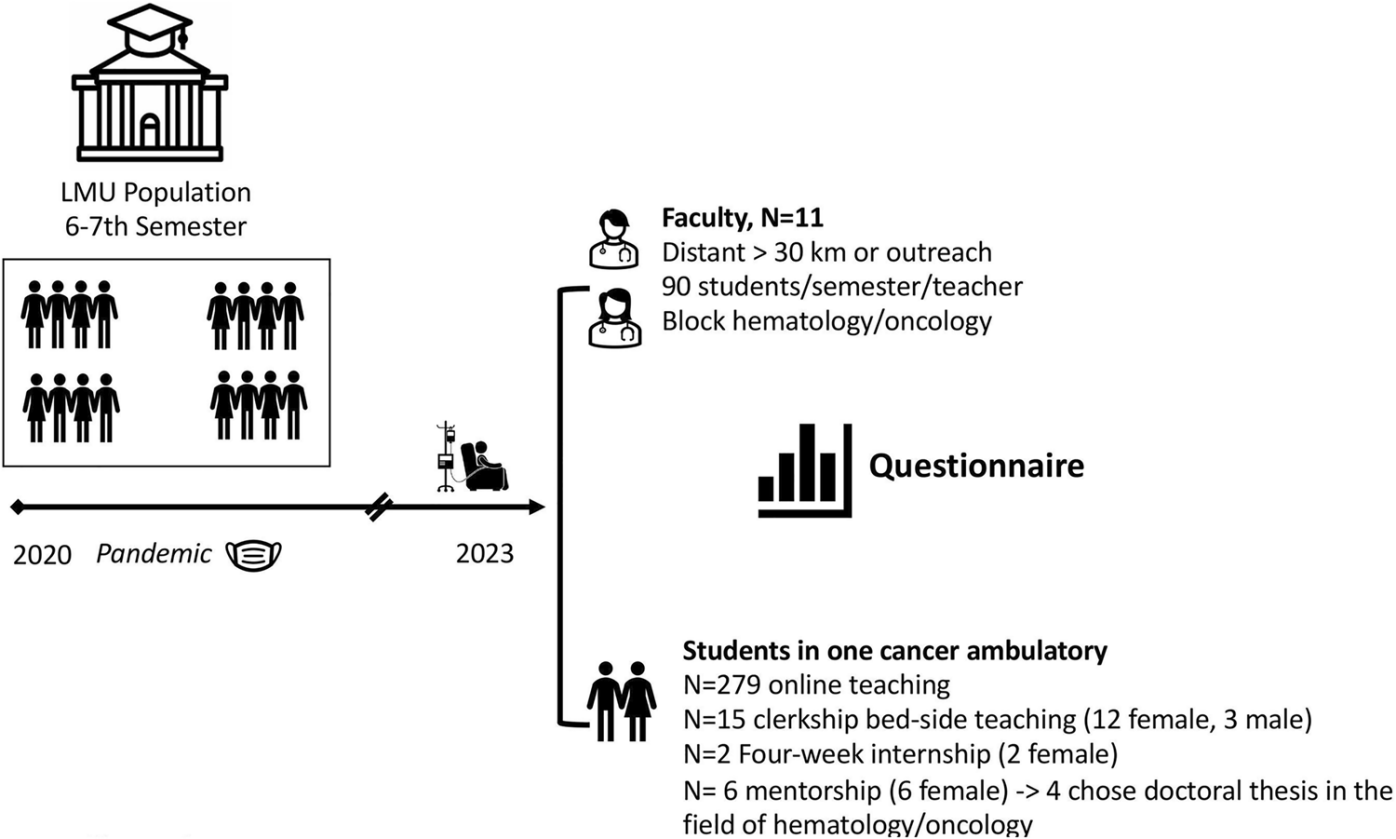
Study population and design: Evaluations from one ambulatory cancer center, including online-lessons, bed-side teaching and mentoring program

Figure 1 summarizes the study population and design: Eleven faculty members from distant (> 30 km) institutions and outpatient cancer centers were teaching an average of 90/students/semester in their courses. The evaluation mainly focuses on one selected outpatient cancer center about 30 km from Munich, associated with the cancer center of Dachau.

Initially, an adapted survey of 279 students on medical education in outpatient setting during the pandemic from winter semester 2020 to 2021 for online teaching was analyzed. Furthermore, a one-day bed side teaching at this outpatient cancer center institution was offered and integrated in the course after the pandemic on a voluntary base for 15 students and evaluated in a second survey. In the following, two students completed a 4-week internship there, and six students chose an oncological mentorship resulting in four students choosing a doctoral thesis in the field of hematology and oncology (Fig. 1).

### Teaching and evaluation methodology

Online lessons via Zoom were structured upon 90 min to achieve learning goals and competency in interviewing, discussion of differential diagnoses, and outlining diagnostic workflow. Interviews on symptoms were given upon a self-made short movie either by simulation with help of nurses or real-life with selected and informed patients that formally agreed to attend an online lesson. Interactive discussion of further learning goals was conducted with help of the “chat” function, intercalated by multiple choice questions.

All surveys conducted were created and evaluated using the software “evasys” (evasys GmbH, Lüneburg, Germany). Established questionnaires from the semester evaluations of the LMU-medical faculty for online bed-side teaching were offered to all attendees on a voluntary base. (Likert-scaled items from 1 to 6 best to worse) was asked on: 1. Organization and technique (2 items); 2. Learning content (4 items); 3. Didactics and supervision (5 items); 4. Learning success (3 items); 5. Overall ranking (1 item) and 6. Open comments on positive and negative aspects of the lessons as well as further proposals were collected additionally from the participating students (results displayed in Tables 1, 2)

Furthermore, this analysis comprehends the evaluation of the surveys from eleven lecturers working in an ambulant oncological setting or distant to academic institutions (> 30 km) participating in the same online teaching course. Lecturers were questioned on feasibility and technical aspects and on outcome of online lectures.

After the SARS-CoV-2 pandemic, a one-day clerkship on voluntary base was offered to medical students of the LMU-University. Fifteen students (12 female and three male) of the 6th and 7th semester applied for a full-day (8 am to 5 pm) clerkship. The students attended the one-day clerkship in small groups from 1 to a maximum of 3 students/day. Two medical students attended a four-week internship, and six students asked for a mentorship in oncology. Interprofessional clerkship was offered singularly across all functional units like therapy-room, hematological quick laboratory, ultrasound diagnostic and interview with patients and familiars during routine care.

Adapted new evaluation surveys on the evasys online evaluation platform of the LMU were created and offered to those students participating in the voluntarily clerkship, asking about previous contact of the students to the field of oncology as well as their benefits from the one-day bedside teaching. Furthermore, learning objectives from the national catalog of learning objectives in medical school (Becker et al. 2020) were established prior to completing the course and their achievement was evaluated after the course.

Finally, the outcome of the mentoring chosen by six students was evaluated.

### Data analysis

The data was collected and analyzed anonymously. Descriptive statistics such as mean, median, ranges, standard deviations, and frequencies were used to summarize the results and evaluated via the software “evasys” and Microsoft Excel**.** Qualitative content analysis according to Kuckartz was used to evaluate the free text responses; aspects mentioned by five or more students were considered for evaluation and are displayed in Table 2.

## Results

With the beginning of the pandemic in the winter term 2020, the LMU-faculty of medicine redirected bed-side teaching lessons of outpatient and distant faculty members to online teaching. On request, this model continued to be performed up to 2023 for eleven selected faculty members in non academical or distant institutions.

### Evaluation of online teaching in a selected outpatient cancer center

Table 1 summarizes the evaluations of a total of 279 medical students attending online sessions for a total of seven seminars of 90 min with a median of 40 students/lesson (2020–2023) held by a hemato-oncology specialist from an outpatient cancer center. A total of 102 evaluations (response rate 36.6%) with a median of 14/lesson were completed and uploaded for analysis. All single categories on organization, content and didactic as well as the overall evaluation of the online seminars were rated very positively and met the expectations of the students (range of the mean scores for all items 1.2–1.5, Likert-scaled items from 1 to 6 best to worse). The success of the seminar was especially indicated by the mean score of 1.2 on the item “overall evaluation” with a small range of 1.0–1.3 for all answers (n = 102).

**Table 1:** Students’ evaluations on online teaching, based on an established evaluation questionnaire using a Likert-Scale with a score from 1 to 6 (best to worse)

One faculty experience in an outpatient oncological center	LMU-Students, 6th–7th semester@@Modul 23@@hematology and oncology
Online teaching@@ wS 20-WS 22	279, 102 (36%) evaluations
Organization and technique	Mean score 1.2
2 items	Median: 1
	*Range 1–4*
Learning content	Mean score 1.3
	Median: 1
4 items	*Range 1–3*
Didactic and supervision	Mean score 1.3
	Median: 1
5 items	*Range 1–4*
Learning success	Mean score 1.5
3 items	Median: 1
	*Range 1–5*
Overall evaluation	Mean score 1.2
1 item	Median: 1
	*Range 1–3*
	*SD: 0.4*

In the voluntary free comments most of the students appreciated and praised the simulation movies and the online interview with two patients (n = 25, 24.5% of all answers) as well as the choice of “real life cases” helpful for clinical practice and learning an emphatical attitude (n = 16) (Table 2). Also stimulating active and interactive discussions via multiple choice questions, “chat” and the “survey” function were very much appreciated. The students also indicated a good response from the lecturer to questions from the students (n = 12), as well an interactive design of the case studies using many open questions to the students (n = 13), which created a “a motivating and pleasant learning environment”.

**Table 2:** Free text responses on positive and negative feedback regarding the online lessons, evaluated using qualitative content analysis according to Kuckartz. Aspects were included in the table from five or more mentions in all responses.

Positive feedback	Number of mentions in free text responses
Video on the patients ‘ medical history	25
Case-based learning	16
Interactivity	13
Lecturer	12
Multiple choice quiz	11
Live Interview with a patient	10
Negative feedback	
Technical issues when playing videos via Zoom	16
Slides not uploaded to online teaching platform	8

A criticism was moved towards the not optimal transmission of the movies due some technical problems (n = 16) and the slides not being uploaded to the online teaching platform for self-studying (n = 8) (Table 1). The students stated that they wanted to learn more about the aspects of therapy options, radiological findings, microscopic images, and topics in current research. These aspects are already taken into account in the conception of an interdisciplinary one-week course in oncology, which has been newly established in the curriculum of the clinical study section at the LMU (https://medizinische-fakultaeten.de/themen/studium/nklm-nklz/).

Five of the participating lecturers stated that online teaching could be integrated well into their everyday schedule and agreed with the statement that they were able to reach more students with online teaching compared to previous regular semesters (Suppl. Figure 1). There were mixed opinions on whether online teaching allowed as much student interaction as face-to-face teaching as well as if more lessons could be provided due to online lessons.

### Evaluation of bed side teachings and clerkships in a selected outpatient cancer center

Fifteen students attended the one-day clerkship. 10 students completed the evaluations, resulting in a response rate of 66%. Their profile is summarized in Table 3. Twelve of them were female (80%) reflecting the current trend in medicine and the interest in oncology in the new generation of medical students and 70% were from the 6th–7th semester (currently attending courses in internal medicine and surgery).

**Table 3:** Characteristics and evaluations of the students attending the one-day bedside teaching in an ambulatory setting, based on a newly developed questionnaire (multiple answers possible)

One faculty experience in outpatient oncological center	Participants	Returned questionnaire
One-day bed side teaching	15 (12 ♀, 3 ♂)	10 evaluations (66% response rate)
SS/WS 2022		
SS/WS 2023		
Semester		
Modul 23	70%	
Modul 4	30%	
Previous contact to oncology		
Lessons	100%	
Inpatient Bedside teaching	90%	
Internship	20%	
Others	50%	
Exposure to outpatient care		
Lessons	20%	
Bedside teaching	20%	
Internship	60%	
Others	40%	
Outpatient internship experience		
Yes	40%	
No	60%	
Do you feel this outpatient experience improved your understanding of outpatient practice?		
Yes		90%
No		10%
I feel that from this outpatient experience I improved my understanding in the intersectoral processes		
Yes		90%
No		10%
I wish a regular integration in curriculum of one-day bed side teaching in an outpatient setting in oncology		
Yes		90%
No		10%
Specialty preference for internships		
Outpatient settings (e.g., gynecology, internal medicine)		67%
Emergency room		22%
Don´t know yet		11%
Anesthesiology		0%
Free comments on positive aspects		N = 9
Active interaction with patients		
Overview in practical processes in outpatient care		
Training with sonography		
Free comments on wishes for the one-day bedside teaching		N = 1
Counseling on chemotherapy		

Previous exposure during studies to oncology/hematology was predominantly through frontal lessons and inpatient bedside teaching. Exposure in general to an outpatient care institution was predominantly through internships, only 20% from lessons and bed side teaching. More than half (60%) of the students surveyed had no previous outpatient internship experience.

Table 3 furthermore points out the results of the survey on the improvement in learning the outpatient and intersectoral practice. The majority of students (90%) stated an improvement in understanding of outpatient practice as well as intersectoral processes due to the one-day bedside teaching and favored an integration of this new teaching format into the regular medical curriculum. As in Germany an ambulatory internship is mandatory in medical studies, two thirds of the participating students preferred the internship in an outpatient setting, and only 22% in the emergency department/ 0% in anesthesiology.

The answers in the free comments in the survey showed a high degree of appreciation and satisfaction for the opportunity of a clinical, interprofessional and multidisciplinary experience including training in sonography and for the unformal and near contact to a considerable number of patients and familiars, improving their motivation to studying. Two students applied for a 4-weeks clerkship in the ambulatory center.

Further six students applied for the LMU-mentoring program (Oettle et al. 2023) and four of them opted for doctoral thesis in hematology/oncology after attending the clerkship.

Table 4 shows the subjective improvement of students in terms of specific competencies of the NKLM. Unlike other courses, the one-day bedside teaching allows the imparting of competencies of many different fields of competences, such as therapeutic measures, medical interviewing, interprofessional competences, health counselling, leadership and management, and clinical-practical skills; in this case all with a focus on oncological patient care. The most improvement in knowledge was indicated in the competences regarding medical interviewing (especially setting up a joint diagnosis and treatment plan, mean score 1.2 indicating a considerable improvement in knowledge, Likert-scaled item 1—agree strongly to 5—strongly disagree) as well as interprofessional competences (especially regarding the work of medical assistants, mean score 1.3) (Table 4). The least improvement in knowledge was evaluated with regard to leadership and management competencies (mean item scores 2.4 and 2.2, median scores 1.5 and 2.5, Table 4).

**Table 4:** Previously determined learning objectives from the NKLM 2.0; students were asked if they improved knowledge regarding those according to prior knowledge. Rating from 1—agree strongly to 5—strongly disagree; evaluated were mean, median, range and standard deviation (sd)

One faculty experience in outpatient oncological center, evaluation of learning objectives (“competences” from NKLM 2.0)	Survey, n = 10
The student is able to…	
Therapeutic measures	
… explain the principles of curative, adjuvant, neoadjuvant, and additive therapy approaches using relevant examples and critically discuss them in the context of clinical trials	Mean: 1.7 Median: 2 Range: 1–3 sd: 0.7
… name and explain the basic pharmacological principles of therapy of common malignant tumours with their drugs	Mean: 1.8 Median: 2 Range: 1–3 sd: 0.6
Medical interviewing	
… conduct and document a patients’ history appropriate to the situation	Mean: 1.5 Median: 1 Range: 1–4 sd: 1.0
… deliver bad news appropriately with situation-specific consideration of a conversation model	Mean: 1.8 Median: 1 Range: 1–4 sd: 1.1
… discuss, determine, and modify as needed a joint diagnostic and treatment plan	Mean: 1.2 Median: 1 Range: 1–2 sd: 0.4
Interprofessional competences	
… explain the duties, function, and responsibilities of medical assistants	Mean: 1.3 Median: 1 Range: 1–3 sd: 0.7
… provide a target group oriented, correct and understandable documentation of information about the treated person and about his/ her individual situation	Mean: 1.7 Median: 1 Range: 1–3 sd: 0.9
… develop individually relevant therapy and rehabilitation goals (interprofessionally as well as together with the patients and, if necessary, their relatives)	Mean: 1.4 Median: 1 Range: 1–3 sd: 0.7
Health counselling, promotion, prevention and rehabilitation	
… understand epidemiologic measures and communicate them in patient-friendly language	Mean: 2.2 Median: 2 Range: 1–4 sd: 1.1
Leadership and management competences	
… derive and reflect on areas of tension in the normative and social context for physicians (for example, ethics in research, compulsory vaccination, tension between medicine, quality, ethics and economics)	Mean: 2.4 Median: 1.5 Range: 1–5 sd: 1.7
… name and describe economic principles (incentive systems) and their significance for the physician's own actions	Mean: 2.2 Median: 2,5 Range: 1–4 sd: 1.1
Clinical-Practical Skills	
… conduct a survey of lymph node status	Mean: 1.9 Median: 1,5 Range: 1–5 sd: 1.3
… examine of the female breast and its lymph node stations	Mean: 2.3 Median: 2 Range: 1–5 sd: 1.6
… handle of port systems and central venous access in compliance with the indication, risks and hygiene measures	Mean: 2.1 Median: 1,5 Range: 1–4 sd: 1.4

Of note is furthermore, that the two selected patients that agreed to be interviewed for online teaching and most patients involved in the students´ mentorship in a traditional “non- academic” but rather in a familiar primary care environment, were very honored and encouraged students as well as the teaching staff. Also, the patients expressed their feeling to receive a qualitatively better care from treating physicians and nurses during students’ clerkships.

### Mentoring program encourages medical students regarding medical studies and research in the field of hematology and oncology

Table 5 analyses the outcome of the mentoring program. Five of the six students had previously completed the one-day clerkship and two participated in the four-week clerkship.

**Table 5:** Evaluation of mentorship in hematology/ oncology (multiple answers possible)

Outcome of mentoring	Evaluation of mentoring
LMU-mentees of the program LMU-Mentoring	6 (6 female, 0 male)
(6 th–7th semester)	
One-day clerkship	5
Four-week clerkship	2
Doctoral thesis in hematology/oncology at LMU	4
Pursue/completion	2 (pathology, genetic)
Initiation	2 (Gyn-oncology, Leukemia)
Cancer-related area of research	
Basic research	2
Clinical research	2
Chose a cancer-related discipline for future	
Attended scientific meetings	4
Publication (congress, journal)	> 2

Four female students initiated or completed a PhD thesis in the field of hematology and oncology in basic research and clinical studies respectively with attendance to scientific meetings. To date, two students succeeded in abstract publication in scientific meetings and submitted their manuscripts to peer-reviewed journals.

## Discussion

This observational longitudinal study on teaching in the field of hematology and oncology in an ambulatory setting started with the necessity of shifting face-to-face bedside teaching into online teaching with the emergency of the pandemic. On request of faculty members of outpatient care centers and institutions at long distance this model was prolonged by virtue of being more effective and time/cost saving. At present, this model has been established, and it is now fully integrated in the curriculum.

The current survey of this pilot project with focus on an outpatient primary cancer care setting on the online learning/ as well a voluntary one-day internship demonstrates its feasibility and reveals very positive feedback on content and didactic methodology. Furthermore, the students reported an improvement in knowledge regarding the learning objectives from NKLM 2.0 due to the one-day clerkship (Table 4). The results of the students’ evaluations show that this model is feasible despite social or geographical distancing restrictions and delivers high quality teaching, improves self-reported knowledge on ambulatory medicine and interprofessional care resulting in satisfaction and wellbeing of students and patients. Finally, this model creates the optimal frame for longitudinal mentoring of medical students and improves motivation and the ethical attitude towards subspecialties.

Especially in the field of hematology/oncology there is a tremendous need for more specialists in the ambulatory setting nationwide as the number of patients as well as the variety, numbers and duration of new therapeutic agents are developing exponentially. On the other hand, after the pandemic there was a significant decrease in the percentage of young physicians choosing oncology as a specialty and of young oncologists changing their direction due to the high burden of stress and responsibility. Hence, this pilot project may suggest a model to integrate outpatient care teaching in several subspecialties in the near future.

There are several advantages of an outpatient setting education: 1. the chance to widen the spectrum of teaching to more subspecialties rather than general medicine like hematology and oncology or other internal/surgical specialties, that, in the German health system, are practiced in the vast majority of patients in ambulatory setting; 2. The chance to improve motivation and education outcomes by smaller groups of students, more individualized and possibly longitudinal mentoring; 3. The chance to teach and learn in a high-efficient, independent, and familiar environment; 4. The chance to involve more academic staff working in outpatient setting improving the bridge of clinical care and research between academic and the real-world ambulatory institutions.

Still, the development of programs for ambulatory training in today´s healthcare environment has many barriers and challenges: A comprehensive review of medical education literature identified both human and structural factors that may negatively influence academic goals in outpatient medical education (Oliveira Franco et al. 2019). The main barriers are related to educational environments and institutions e.g., lack of institutional support, conflicts between medical education and healthcare; outpatient and university curricula not being integrated; inadequate financial incentives for academic staff; but also, academic staff-related like lack of preparation or professional training and inadequate service model for students. Barriers are also patient-related like lack of suitable patients or failure to obtain consent or no-follow-up. Finally, a major concern for both students and teachers is the increasing number of students in internship groups (Oliveira Franco et al. 2019; DAM, LMU, MeCuM 2007).

In Germany, integration of outpatient education in the curriculum is partly considered for general medicine as pointed in the “masterplan medical education 2020”; however, for a structured implementation—in particular for subspecialties—it is necessary to create a legislative framework for cooperation between medical faculties and outpatient healthcare systems (Becker et al. 2020).

Limitations of our study include the use of non-validated questionnaires and no measurable feedback (evaluation forms) from patients and nurses. The response rate of 36.5% (102 out of 279 students) for the evaluations of online lessons, as well as the response rate of 66.6% (10 out of 15 students) for those students attending the one-day clerkship, indicates a representative sample of student feedback. However, it is important to acknowledge certain limitations when interpreting these results. The rather small sample size (especially regarding the non-online lessons), alongside the unicentric nature of the data collection, restricts the generalizability of the findings. These factors suggest that the conclusions drawn from this study should be viewed with caution, particularly when considering their applicability to a broader, more diverse cohort of medical students or institutions. Nevertheless, this study serves as a pilot project aimed at exploring the integration of outpatient oncology teaching into the medical curriculum. Given the preliminary nature of this work, further research involving larger and more diverse samples, as well as multicentric data collection, is essential to validate our findings.

### Perspectives

Because enhancing ambulatory education has high priority in graduate medical education, we promote the continuation and expansion of this model upon licensing regulations and support by academic institutions. In the upcoming semesters, the teaching by outpatient oncologists is planned to be further developed and systematically monitored as a process and chance for future medical education in Germany.

## Supplementary Information

Below is the link to the electronic supplementary material.

Supplementary file1 (DOCX 17 KB)

## Data Availability

No datasets were generated or analysed during the current study.
