# Supplementary material for: Hemato-oncological outpatient care in medical education: a German pilot-project
Source: J Cancer Res Clin Oncol. 2025 May 20;151(5):172. doi: 10.1007/s00432-025-06198-7 (PMC12092479; doi:10.1007/s00432-025-06198-7)
Supplement: Supplementary file 1 — Supplementary file1 (DOCX 17 KB) [file 432_2025_6198_MOESM1_ESM.docx]

Supplementary Figures

1

| Evaluation | LMU-faculty member  hematology and oncology |
| --- | --- |
| Lecturers, WS 20-WS 22  Outpatient oncological care < 30 km  Hospitals, hem/oncology > 30 Km  Outpatient oncological care > 30 km  Response to survey | 11  4  3  4  5 (45%) |
| In the WS 20-WS 22 I was able to offer more courses compared to face-to-face semesters. | Mean 2.6  Range 1-5  sd 2.2 |
| In the WS 21-WS 22 I was able to reach more students with my lesson compared to face-to-face semester. | Mean 4.8  Range 4-5  sd 0.4 |
| The online teaching integrates well into my daily practice routine. | Mean 4.8  Range 4-5  SD 0.4 |
| There were technical problems before or during lessons. | Mean 2.0  Range 1-4  sd 1.4 |
| The online teaching offered equal student interaction compared with the face-to-face lessons. | Mean 3.6  Range 2-5  sd 1.1 |
|  |  |

**Fig. 1:** Lecturers’ evaluations on online teaching, based on a newly developed questionnaire using a Likert-Scale with a score from 1 – 5 (Strongly disagree to agree), using mean, range and standard deviation (sd).
